# Plants and microplastics: Growing impacts in the terrestrial environment

**DOI:** 10.3389/fpls.2025.1666047

**Published:** 2025-09-30

**Authors:** Amanda E. Wong, Gail Taylor

**Affiliations:** ^1^ Department of Plant Sciences, University of California, Davis, Davis, CA, United States; ^2^ Department of Genetics, Evolution and Environment, University College London, London, United Kingdom

**Keywords:** microplastics, pollution, terrestrial plants, stress responses, plastic uptake

## Abstract

Microplastic pollution is a largely unexplored yet pervasive environmental problem, in terrestrial environments, including impacts on plants and food crops. Plant growth and function are most often negatively impacted by plastic exposure, but these pollutants can also stimulate plant processes such as root growth and there is a tentative suggestion that monocotyledonous may be less sensitive to microplastics than dicotyledonous plants. Toxic effects include reduced plant biomass, chlorophyll content, photosynthesis, and changes to antioxidants, metabolites, and nutrients, with stimulatory effects often found at lower concentrations of exposure. There is strong evidence that roots can directly uptake and translocate plastic particles at 1 µm and under in size. Indirect effects include interactions of microplastics with other pollutants, soil properties, and soil organisms. These findings have potentially wide-ranging implications for terrestrial ecosystem function and human health. Future research should further elucidate the mechanisms of plant microplastic toxicity at realistic concentrations. This short review highlights the significance of microplastics in the terrestrial environment, where they can occur at higher concentrations than in the aquatic environment, with likely impacts on important food crop plants. The significance of these findings for human and ecosystem health remains to be elucidated and we make four recommendations to the scientific community for improved future experimentation.

## Introduction

Studies on the occurrence and effects of microplastics and smaller nanoplastics have been increasing, with a major focus on aquatic ecosystems, but recent plastic pollution research on terrestrial plants and ecosystems is emerging (Rillig, 2012; de Souza Machado et al., 2018a; Zhou et al., 2021). Since the 1950s, approximately 8,300 million metric tons of plastic was produced (Geyer et al., 2017), and rising global plastic consumption has resulted in increased plastic waste and widespread presence and persistence in the environment (Thompson et al., 2004; Jambeck et al., 2015). Plastics are polymers [e.g. low-density polyethylene (LDPE), high-density polyethylene (HDPE), polystyrene (PS)] that also contain a variety of chemical additives, such as plasticizers, pigments, and flame retardants (Geyer et al., 2017), making them a diverse pollutant group, with respect to chemical composition, size, shape, concentration, and source (**Supplementary Figure S1**). Plastic pollution originates from the direct release of plastics and secondarily from the fragmentation of larger plastics (Horton and Dixon, 2017). In the environment, plastics degrade into smaller plastic fragments from exposure to ultraviolet radiation, heat, and water. As plastics fragment, the surface area to volume ratio increases along with the bioavailability of these particles, potential to leach chemical additives, and ability to accumulate other pollutants in the environment (de Souza Machado et al., 2018a).

**Figure 1:**
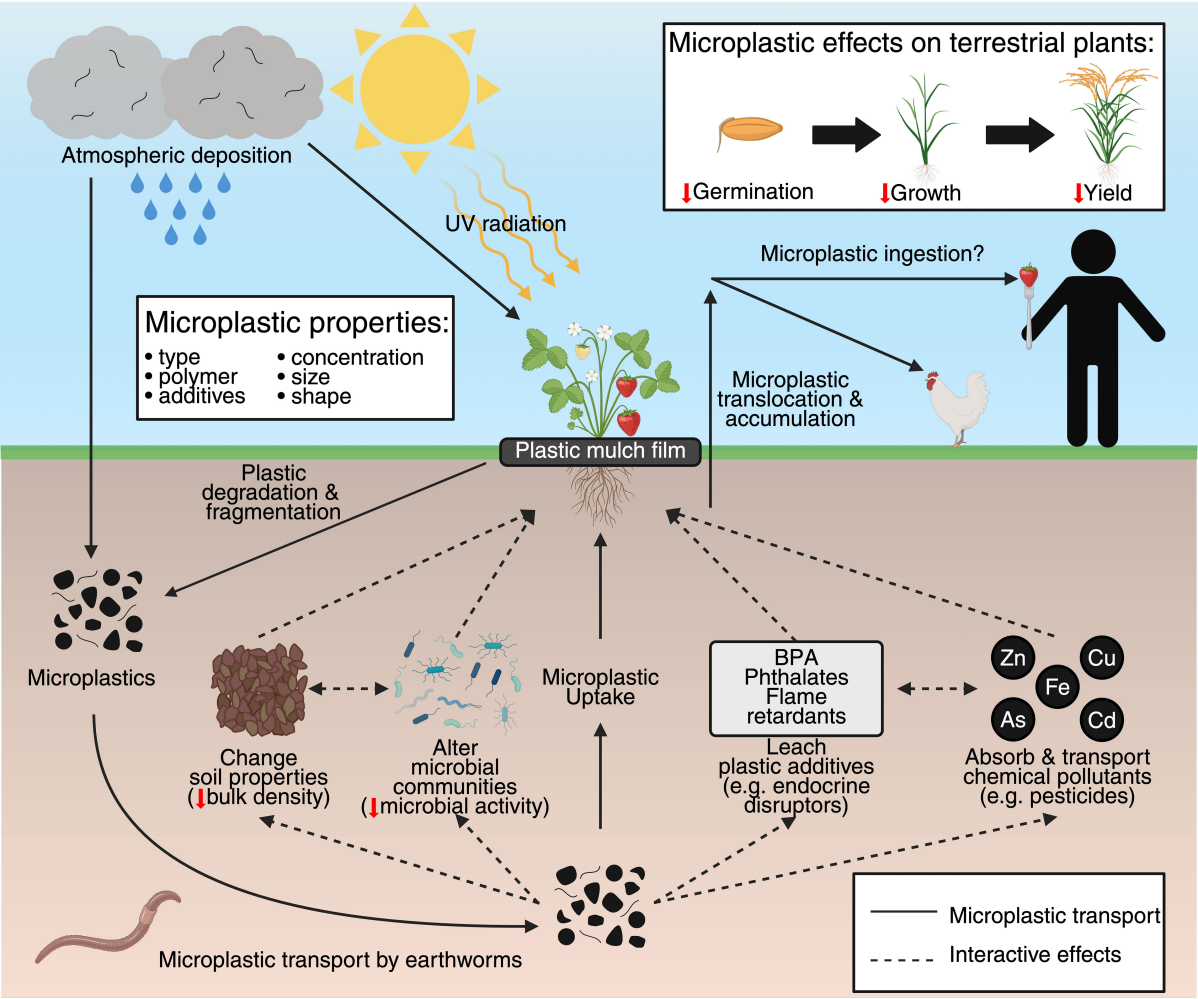
Pathways of microplastic transport in terrestrial environments and direct and indirect effects of microplastics on terrestrial plants. Plastics enter terrestrial ecosystems through the direct application of plastic products, such as plastic mulch film in agriculture and horticulture. In the environment, ultraviolet radiation exposure degrades and fragments plastics to form microplastics, while microplastics also enter terrestrial ecosystems through other pathways, such as atmospheric deposition (Horton et al., 2017). In the soil, microplastics are transported throughout the soil profile by soil organisms, such as earthworms (Huerta Lwanga et al., 2017a; Rillig et al., 2017). Experiments have demonstrated microplastic uptake, translocation, and accumulation by terrestrial plants (Li et al., 2020; Dong et al., 2021b; Liu et al., 2022), while food chain studies provided evidence of microplastic trophic transfer (Huerta Lwanga et al., 2017b; Abdolahpur Monikh et al., 2022), which suggests potential microplastic ingestion by humans. Microplastics interact with soil properties (de Souza Machado et al., 2019), microorganisms (Fan et al., 2022), plastic additives (Pflugmacher et al., 2021), other chemical pollutants (Zong et al., 2021), and one another to indirectly affect terrestrial plants. Microplastics negatively impact terrestrial plants across multiple developmental stages, from germination to growth to reproduction and, ultimately, crop yield (Wu et al., 2020). (BPA), bisphenol A; (UV), ultraviolet; (Zn), zinc; (Cu), copper; (Fe), iron; (As), arsenic ;(Cd), cadmium. Created with BioRender.com.

On land, plastic pollution is released from urban, industrial, and agricultural settings (Horton and Dixon, 2017). Microplastics enter agricultural soils directly from horticultural and agronomic usage and fragmentation of plastic mulching, greenhouse materials, irrigation pipes, and packaging and indirectly from contaminated compost, treated wastewater and sewage sludge, surface runoff, and atmospheric deposition (Scarascia-Mugnozza et al., 2012; Horton et al., 2017; Ng et al., 2018). Horton et al. (2017) estimated that plastics are released to the terrestrial environment at 4–23 times more than that in the marine environment, highlighting the threat to terrestrial ecosystems and plants, but despite this, there is limited research on plastic pollution impacts on plants (Rillig et al., 2019). Here we discuss the direct impacts of diverse microplastics on terrestrial plants at different development stages and the interaction between microplastics and the broader terrestrial ecosystem that indirectly affects plants (**Figure 1**). We propose a conceptual model for how microplastics impact plants and highlight the need to elucidate the mechanisms of action of microplastics on plants to understand how realistic microplastic exposure conditions impact terrestrial plants.

## Uptake and translocation of plastics

Recent studies have identified plastic particle uptake, translocation, and accumulation in terrestrial plants, while plants were assumed to be unable to uptake larger plastics since particles bigger than approximately 5–20 nm are unable to enter cell walls (Schwab et al., 2016). However, even 50 nm plastics were found in the vacuoles and cytoplasm of onion (*Allium cepa*) root cells (Giorgetti et al., 2020), while 1,000 nm plastics can accumulate in the intercellular space of rice (*Oryza sativa* L.) and carrot (*Daucus carota*) roots and shoots (Dong et al., 2021b; Liu et al., 2022). In lettuce (*Lactuca sativa* L.) and wheat (*Triticum aestivum* L.), 200 nm particles were found in the roots, shoots, and leaves, such that the particles were translocated from the roots to the shoots through transpiration that pulled strings of plastic particles throughout the vascular system (Li et al., 2020). Both 200 nm and 2,000 nm particles entered the root stele at discontinuous and developing areas of the Casparian strip where lateral roots emerge, while more plastics accumulated in hydroponic plants compared to sandy soil as a result of weaker root apoplastic barrier and higher transpiration (Li et al., 2020). Interestingly, only negatively charged plastic particles were detected in the apoplast and xylem in *Arabidopsis thaliana*, while positively charged particles stimulated exudate production that trapped the plastics and reduced uptake (Sun et al., 2020). However, one study found no evidence of plastic uptake in wheat and *A. thaliana* (Taylor et al., 2020). Another recently discovered pathway for microplastic uptake is leaf adsorption via accumulation on trichomes, stomatal uptake, and apoplastic transport (Li et al., 2025), which highlights the ubiquity of microplastics in the environment and the persistent exposure of terrestrial plants to these pollutants.

**Figure 2:**
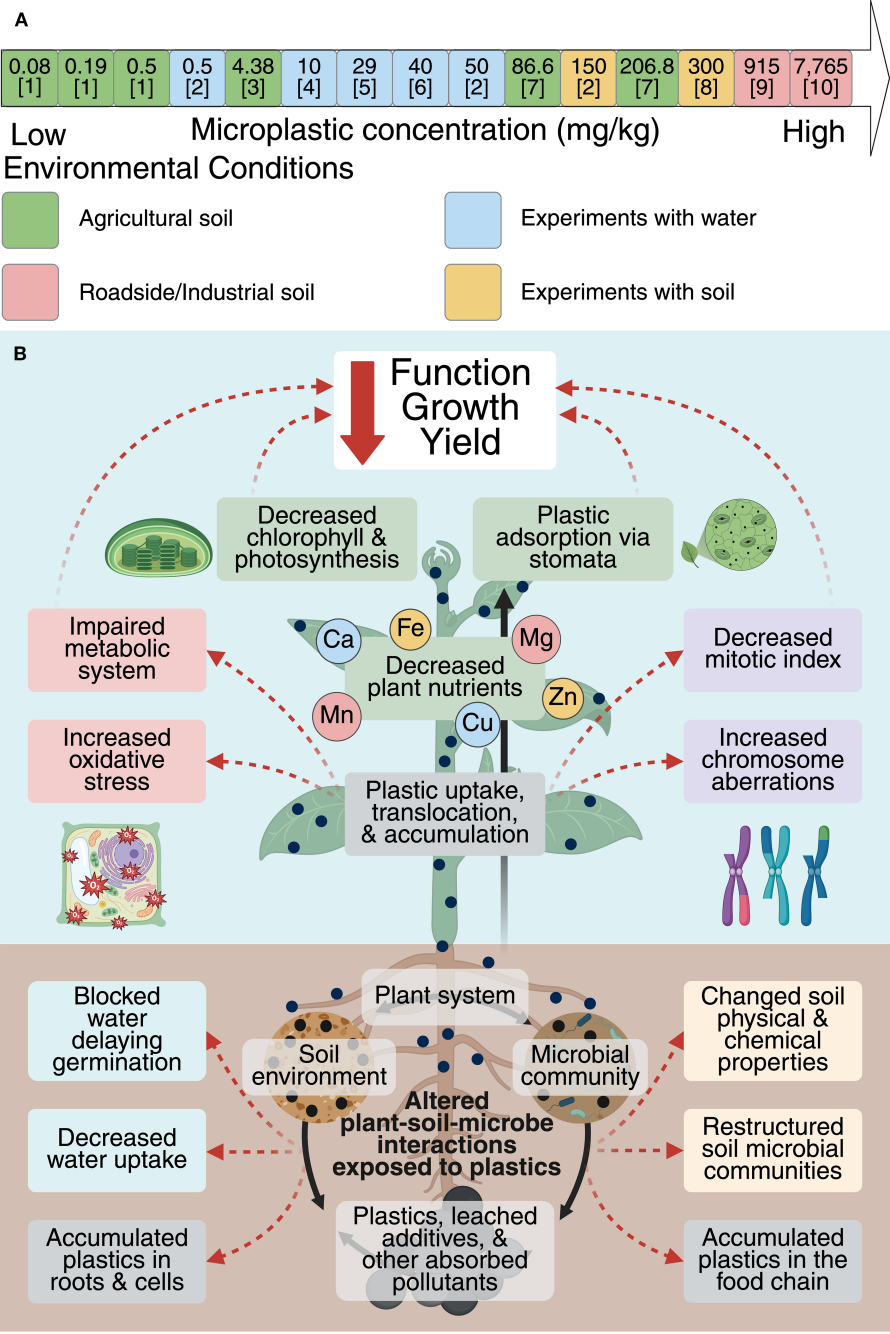
Plant exposure to microplastics & a conceptual model of the impacts of microplastics on plants. Figure **(A)** represents microplastic concentrations in the soil environment vs. experiments. Microplastic concentrations in plant exposure experiments with test solution (blue) or soil matrix (yellow) compared to microplastic concentrations found in agricultural soil (green) or roadside/industrial soil (red), where the microplastic concentration (mg/kg) is reported, followed by the corresponding reference in brackets within each colored square. The microplastic concentrations in plant exposure experiments in blue and yellow boxes are the studies discussed in the microplastic uptake section: [2] (Li et al., 2020), [4] (Dong et al., 2021b), [5] (Taylor et al., 2020), [6] (Liu et al., 2022), [8] (Sun et al., 2020), which depicts the lowest microplastic concentrations that the plants were exposed to in the microplastic uptake studies. The microplastic concentrations found in the terrestrial environment in green and red boxes are mean or median microplastic concentrations in soil from agricultural fields: [1] (Zhang et al., 2018), [3] (Corradini et al., 2019), [7] (Radford et al., 2023), roadsides [9] (Dierkes et al., 2019), and industrial settings [10] (Fuller and Gautam, 2016). Created with BioRender.com. Figure **(B)** represents a conceptual model of the impacts and mechanisms of action of microplastics on plants, from the current literature. In the soil, microplastics physically change the soil but also leach plastic additives and absorb other pollutants that impacts soil chemical properties, the soil microbial community, and plant-soil interactions (de Souza Machado et al., 2018b, 2019), with implications for plant water and nutrient uptake. Plants, including food crops uptake, translocate, and accumulate microplastics through the vascular system and into plant cells (Li et al., 2020). Microplastic exposure induced physiological changes, cytotoxicity, genotoxicity, and decreased nutrient content in plants and food crops (Giorgetti et al., 2020; Urbina et al., 2020; Colzi et al., 2022). Ultimately, microplastic exposure and uptake resulted in decreased function, growth, and yield in plants, including food crops (Wu et al., 2020). Seed germination is also sensitive to microplastics with both stimulation and inhibition observed with a tendency for positive effects to be seen in grasses. Abbreviations: zinc (Zn); copper (Cu); iron (Fe); calcium (Ca); magnesium (Mg); manganese (Mn). Created with BioRender.com.

The results of these studies demonstrated that plants can directly take up microplastics with major implications for terrestrial plants and crop quality and potential human ingestion and health impacts. However, it is important to consider the relevance of the concentration of microplastics and type of media used in such uptake experiments and how they relate to microplastic concentrations and terrestrial plant exposure in the wider environment (Fuller and Gautam, 2016; Zhang et al., 2018; Corradini et al., 2019; Dierkes et al., 2019; Radford et al., 2023). Overall, concentrations in microplastic uptake experiments are reasonable but tend to be at the higher end of the spectrum of environmental exposure, especially for soil-based experiments, and this could bias the results observed (**Figure 2A**). Future research focused on microplastic uptake by terrestrial plants exposed to lower microplastic concentrations in soil would be of value, especially for food crops.

## Germination and seedling development

Our conceptual model shows that multiple studies investigated the effect of microplastic exposure on germination and seedling development, with the majority demonstrating a negative effect of microplastics on the early stages of plant development (**Table 1A**). Seed germination rate generally decreased significantly following exposure to microplastics as the concentration and size increased, where the microplastics accumulated on the seed pores, blocked water, and delayed germination (Bosker et al., 2019; Guo et al., 2022). However, several studies found negligible differences in germination after 24 hours, which may be attributed to the nano-priming effect of small microplastics that promoted seed germination (Lian et al., 2020; Zhang et al., 2021, 2022; Shorobi et al., 2023). During seedling development, exposure to microplastics frequently inhibited root growth and decreased root length, but not in wheat (Bosker et al., 2019; Jiang et al., 2019; Lian et al., 2020; Bao et al., 2022; Iswahyudi et al., 2024). Microplastic exposure induced cytotoxicity and genotoxicity in seedling roots through a decrease in mitotic index and an increase in chromosome aberrations as microplastic concentration and time increased (Gopinath et al., 2019; Jiang et al., 2019; Giorgetti et al., 2020; Maity et al., 2020). Although not conclusive, we propose a tentative hypothesis from the data available, that monocotyledonous species may be less sensitive to microplastics than dicotyledonous species, given impacts on seed germination. As a working hypothesis this is useful but requires further research to confirm or reject this proposal.

**Table 1:** The effect of microplastics on plant germination, seedling development, growth, and physiology.

Table 1A Germination and seedling development										
Ref.	Species	Exposure Time	Polymer Type	Size	Shape	Concentration	Germination	Root Growth	Health/toxicity	
Guo et al., 2022	*Trifolium repens, Orychophragmus violaceus*, & *Impatiens balsamina*	7 days	PS	2 µm, 80 nm	Fragment	0, 10, 50, 100, 500 mg/L	-			
Bosker et al., 2019	*Lepidium sativum* L.	3 days	Polymer	50, 500, 4800 nm	Sphere	10^6-10^10 particles/L	-	-		
Esterhuizen and Kim, 2022	*Nelumbo nucifera*	7 days	PP, PVC, PUR, PET, HDPE, PS	4 mm	Fragment	14% (w/w); 4 g/L	-			
Boots et al., 2019	*Lolium perenne*	30 days	HDPE, PLA, fibers	HDPE: 102.6 µm & PLA: 65.6 µm	Fragment	HDPE & PLA: 0.001% (w/w) & fibers: 0.1% (w/w)	-			
Pflugmacher et al., 2020	*Lepidium sativum* L.	7 days	PC	3 mm	Granule	0.1%, 1.0%, and 10% (w/w)	-			
Pflugmacher et al., 2021	*Lepidium sativum* L.	7 days	PC	3 mm	Granule	2% (w/w)	-			
Lian et al., 2020	*Triticum aestivum* L.	5 days	PSNP	100 nm	Sphere	0.01, 0.1 1.0, 10 mg/L	0	+		
Giorgetti et al., 2020	*Allium cepa*	3 days	PS	50 nm	Sphere	0.01, 0.1, 1.0 g/L	0	-	-	
Zhang et al., 2021	*Oryza sativa* L.	14 days	PS	200 nm	Bead	0.1, 10, 1000 mg/L	0			
Maity et al., 2020	*Allium cepa* L.	3 days	PS	100 nm	Sphere	25, 50, 100, 200, 400 mg/L		-	-	
Jiang et al., 2019	*Vicia faba*	2 days	PS	0.1, 5 µm	Sphere	10, 50, 100 mg/L		-	-	
Gopinath et al., 2019	*Allium cepa*	3, 6, 12, 24 hours	PS & facial scrub	100 nm	Particles	5, 10, 15, 20, 25 µg/mL		-	-	
Bao et al., 2022	*Triticum aestivum* L.	5 days	PE	200 µm	Fragment	800 mg/L		+		

Table 1B Growth and physiology										
Ref.	Species	Total Plant Biomass	Root growth	Shoot growth	Chlorophyll content	Photosynthesis	Antioxidant enzymes	Metabolites		Nutrient content
Boots et al., 2019	*Lolium perenne*		+	-	0					
Lian et al., 2020	*Triticum aestivum* L.		+	+	+	+		-	-	
Yang et al., 2021	*Brassica chinensis* L.	-			-				0	
Urbina et al., 2020	*Zea mays* L. var. Jubilee	0	0	0						
Urbina et al., 2020	*Zea mays* L. var. Jubilee	-	-	-		-			-	
Qi et al., 2020	*Triticum aestivum*	-								
Colzi et al., 2022	*Cucurbita pepo* L.		-	-	-	-			-	
de Souza Machado et al., 2019	*Allium fistulosum*		+						0	
Lozano et al., 2021b	*Daucus carota*		+	+						
Zeb et al., 2022	*Lactuca sativa*		+	-	-		0	-		
Wu et al., 2020	*Oryza sativa* L.		0	-			-	-		
Wu et al., 2020	*Oryza sativa* L.			-						

Green: positive (+); red: negative (-); yellow: negligible differences (0); white: no data. Table **(A)** represents germination and seedling development. Table **(B)** represents growth and physiology. Ref. corresponds to the references in the main text.

## Plant growth, morphology, and yield

Recent experiments have investigated the effect of microplastic exposure on plant growth, morphology, and yield through soil-based and hydroponic experiments (**Table 1B**). Microplastic exposure decreased total plant biomass in Chinese cabbage (*Brassica chinensis* L.) (Yang et al., 2021), maize (*Zea mays* L.) (Urbina et al., 2020), and wheat (Qi et al., 2020). Similar to the negative effect of microplastics on seedling root development, microplastic exposure decreased root and shoot growth, especially in high microplastic concentrations (Urbina et al., 2020; Li et al., 2021; Colzi et al., 2022; Zhang et al., 2024; Riaz et al., 2025). Hydroponic maize in the high microplastic concentration (100 mg/L) had approximately half the root length of maize in the control, with irregular root development and architecture (Urbina et al., 2020). In contrast, several studies found that microplastic exposure increased root and shoot biomass and length (Lian et al., 2020; Lozano et al., 2021b). Exposure to different microplastics stimulated longer and finer spring onion (*Allium fistulosum*) roots but had variable effects on spring onion bulbs, such that polyester fibers doubled the dry mass while polyamide beads nearly doubled the water content compared to the control (de Souza Machado et al., 2019). Several studies found that long-term microplastic and mulch residue exposure decreased crop yield in rice, cotton (*Gossypium* spp.), and maize (Hegan et al., 2015; Wu et al., 2020; Koskei et al., 2021; Yi et al., 2023; Iqbal et al., 2025). Wu et al. (2020) conducted a rice hydroponic experiment and field trial, where shoot biomass decreased by 12.8% and 25.9% when exposed to microplastic concentrations of 50 mg/kg and 250 mg/kg, respectively, which aligns with the decreased shoot biomass as microplastic concentration increased in the hydroponic experiment.

## Plant physiology, metabolism, and nutrient content

Many of these studies also investigated the effect of microplastics on plant physiology, metabolism, and nutrient content, which can have major implications on plant productivity and health (**Table 1B**). The majority of studies found that microplastics decreased chlorophyll content and photosynthesis (Urbina et al., 2020; Yang et al., 2021; Colzi et al., 2022; Nei et al., 2024; Wang et al., 2024; Zhang et al., 2024). Interestingly, hydroponic wheat exposed to PS microplastics had elevated chlorophyll content, net photosynthetic rate, stomatal conductance, and transpiration rate that peaked at 0.1 mg/L of microplastics and decreased in higher concentrations (Lian et al., 2020). In maize, the high concentration (100 mg/L) of HDPE decreased net carbon fixation, stomatal conductance, and transpiration rate, such that net carbon fixation and transpiration were three times lower compared to the control (Urbina et al., 2020).

Several studies demonstrated changes to plant antioxidant defense systems and metabolism, where microplastic exposure significantly altered wheat and rice metabolites (Lian et al., 2020; Wu et al., 2020). Lettuce exposed to 0.1% microfibers changed 14 out of 46 identified metabolites that increased to 17 altered metabolites under 0.2% microfibers (Zeb et al., 2022). As microplastic concentration increased, rice and tomato (*Lycopersicon esculentum* L.) antioxidant enzyme activity decreased but increased in wheat (Wu et al., 2020; Shi et al., 2022; Riaz et al., 2025). Increased MDA content in tomato and wheat indicated increased oxidative stress while rice had ten inhibited main metabolic pathways, which can lead to decreased growth and crop yield (Wu et al., 2020; Shi et al., 2022; Riaz et al., 2025).

Microplastic exposure also impacted plant carbon, nitrogen, and nutrient content, where high microplastic concentrations decreased shoot nitrogen content in maize but increased in wheat (Lian et al., 2020; Urbina et al., 2020). Interestingly, spring onion exposed to polyamide microplastics had higher foliar nitrogen content, which was likely attributed to the chemical composition of the polyamide beads that released nitrogen into the soil (de Souza Machado et al., 2019). Similarly, Urbina et al. (2020) estimated that maize exposed to HDPE microplastics absorbed approximately 30% of the carbon from HDPE-derived carbon in the maize roots, but not the shoots. As microplastic concentration increased, soluble sugar and protein decreased in tomato while foliar soluble sugar increased but starch decreased in Chinese cabbage (Yang et al., 2021; Shi et al., 2022). Microplastic exposure decreased carotenoid and flavonoid content in tomato fruit and generally reduced micronutrient content in tomato, wheat, and field pumpkin (*Cucurbita pepo* L.) (Lian et al., 2020; Colzi et al., 2022; Nei et al., 2024; Emenike et al., 2025). Although the majority of experiments demonstrated a negative effect of microplastic exposure on terrestrial plant growth, physiology, and traits, there were also a significant number of studies where growth and metabolism were stimulated, particularly for root growth. These differences in the effect of microplastics on terrestrial plants may be attributed to multiple factors, including species, growth medium, microplastic concentration, polymer, size, shape, exposure duration, and/or environmental factors. In Lozano et al. (2021b), the majority of the variance in shoot and root biomass was explained by microplastic polymer, shape, and their interaction, while microplastic concentration alone explained very little of the variation (Lozano et al., 2021b). Therefore, the inherent variability in microplastic characteristics, in addition to different experimental designs, highlights the complex interactions of microplastic pollution on terrestrial plants and where further research to elucidate mechanisms of action of microplastics on plants is warranted (**Figure 2B**).

## Interaction of microplastics with other chemical pollutants

Microplastics themselves not only affect plant growth and traits directly but also have the potential to interact with chemical additives and other environmental pollutants that can further impact plants indirectly. Degrading plastics leach chemical additives over time that contribute to the impact of microplastics on plants. Garden cress (*Lepidium sativum* L.) exposed to polycarbonate (PC) granules and leachate from new and artificially aged PC decreased germination and seedling length as concentration increased (Pflugmacher et al., 2020). As the PC age increased, the magnitude of the negative effect on garden cress growth and chlorophyll content decreased (Pflugmacher et al., 2021). Since bisphenol A (BPA) is known to leach from PC, garden cress was also exposed to BPA that had an intermediate negative effect on seedling growth that was between the severity of new and aged PC treatments, which suggests that the negative effect of new PC leachate might be attributed to a variety of leached chemical additives (Pflugmacher et al., 2020, 2021). Phthalate esters are also common plastic additives that can leach into the environment and disrupt the human endocrine system. Elevated levels of phthalates were found in vegetables, especially leafy vegetables, grown with plastic mulch and greenhouses, which highlights the potential human health implications of terrestrial plastic pollution (Du et al., 2009; Wang et al., 2015; Chen et al., 2017).

In the environment, microplastics also interact with other chemical pollutants, such as heavy metals, that can have a negative effect on plant growth, biomass, and photosynthesis (Dong et al., 2020, 2021b, 2022; Zeb et al., 2022). Carrot co-exposure to PS microplastics and arsenic increased microplastic internalization occurrence and particle size in the intercellular space and within cells because arsenic exposure altered root cells (Dong et al., 2021b). Carrot arsenic content increased with arsenic concentration, but the presence of PS decreased carrot arsenic content (Dong et al., 2021b). Similarly, wheat exposed to copper, cadmium, and microplastics had lower heavy metal content, while increased concentrations of microplastics decreased rice arsenic content, which suggests that microplastics can adsorb heavy metals and, therefore, reduce the uptake of heavy metals by plants (Dong et al., 2020, 2022; Zong et al., 2021). However, microplastics can absorb and desorb heavy metals, which suggests that microplastics can absorb, transport, and desorb other chemical pollutants (Abbasi et al., 2020). For example, wheat co-exposed to microplastics and oxytetracycline, a common antibiotic in manure, resulted in altered antioxidant enzyme activities and plant metabolism (Bao et al., 2022). The evidence is clear that microplastics can interact with chemical pollutants in the environment resulting in increased and perhaps synergistic impacts of pollution on plants.

## Impact of microplastics on terrestrial ecosystems

Microplastics in terrestrial ecosystems can change soil properties, microbial communities, and species interactions, which can indirectly impact terrestrial plants. Soil microplastic pollution can alter soil organic matter, carbon, and nutrients, which impacts plant nutrients (Dong et al., 2021a; Meng et al., 2022). Furthermore, microplastics can alter pH, decrease bulk density, and change the soil structure, such that microplastic fibers with a different shape from typical soil particles caused the greatest changes to soil structure, water holding capacity, and reduced microbial activity (de Souza Machado et al., 2018b, 2019; Boots et al., 2019; Lozano et al., 2021b). Microplastics not only alter the soil environment but also impact the soil microbial community, such that the bacterial and fungal diversity on the surface of microplastics was lower and distinct from the bulk soil (Zhang et al., 2019; Yu et al., 2021; Rillig et al., 2024). Fungal diversity had a stronger response to microplastics than bacteria, but the dissimilarity in the soil bacterial community composition increased as microplastic concentration increased (Fan et al., 2022; Sun et al., 2022). Microplastics affect not only soil microorganisms but also larger soil organisms, such as earthworms that ingest and transport microplastics throughout the soil profile (Huerta Lwanga et al., 2016, 2017a). Even though microplastics negatively affected earthworm biomass, the presence of earthworms alleviated the negative effects of plastic mulch residue on wheat growth and physiology (Qi et al., 2018). However, microplastics can have cascading effects on terrestrial communities through microplastic trophic transfer and biomagnification in natural and experimental food chains, which highlights the major implications of microplastic pollution on terrestrial plants, ecosystems, and humans (Huerta Lwanga et al., 2017b; Abdolahpur Monikh et al., 2022).

## Discussion

Although the threat of plastic pollution is not new, interest in the impacts of plastic pollution on terrestrial plants is increasing because of the wider-ranging potential consequences for crop quality and yield, ecosystem function, and impact on human health (Lozano et al., 2021a; Yates et al., 2021; Mamun et al., 2023; Bethanis and Golia, 2024). Since plastics are expected to accumulate in agricultural soils and degrade faster under a warming climate, it is essential to utilize plastics sustainably whilst reducing plastic pollution, supporting crop production, and protecting ecosystem and human health (Hofmann et al., 2023; Meizoso-Regueira et al., 2024; Wei et al., 2024). However, the physical and chemical diversity of plastic pollution makes it challenging to disentangle their effects on terrestrial plants. There is still no consensus on the type of plastics that may stimulate or inhibit plant processes and a limited understanding of how plant species and genotype can affect these responses.

This review has shown that microplastics can directly and indirectly impact plant growth and function. From the available evidence, our conceptual model shows that the negative effect on plant growth, physiology, and biochemical properties may be attributed to changes in plant-water relations, metabolism, and redox reactions. Microplastic exposure altered plant metabolites and antioxidant enzyme activity, which indicates oxidative stress and damage to the antioxidant defense system and results in a visible reduction in plant growth and yield. It is therefore hypothesized that ion and water relations and water uptake are being impacted through changes in member properties but also water transport through member proteins. However, gaps still remain in our understanding of the mechanisms behind these changes, as most studies focus on investigating microplastic accumulation and distribution in plants and impacts on plant growth without assessing potential changes to plant molecular biology, physiology and metabolism. This is a clear gap in understanding. For indirect effects mediated through chemical interactions, degrading plastics, and wider soil and soil organism interactions, information is needed on how soil and microplastic properties interact to bring about plant uptake and the effects of microplastics on the plant-soil system.

### Future research should work to address the following gaps

**Mechanisms of action:** More detailed information on mechanisms of action is required and the elucidation of how different types of plastic and environments elicit their effects.

**Plastic distribution:** The majority of recent plastic quantification studies in the terrestrial environment are from China and Europe, while the concentration and identity of microplastics in terrestrial ecosystems in other parts of the world are still lacking. A wide-ranging assessment is required to quantify the distribution of global microplastics in terrestrial ecosystems, especially in agricultural systems with different farming practices, including urban and home gardens, enabling more realistic plant exposure experiments.

**Realistic plastics**: Many current experiments fail to use realistic environmental exposure experiments, and the majority of studies utilize pristine rather than fragmented, degraded, and aged microplastics that interacted with other chemical pollutants in the environment. Plastic concentrations, sizes, shapes, aging, and chemical properties should be considered in future exposure experiments, alongside mixtures of other pollutants to ensure a realistic pollution cocktail, including pesticides, fertilizers, and heavy metals.

**Relevant conditions:** Multiple experiments expose terrestrial plants to microplastics in small hydroponic containers for short durations with a single species and a limited number of plant genotypes. In the future, improved experiments should focus on the exposure of soil-grown plants to more relevant environmental conditions, for longer durations and consider natural plant genetic variation, especially diverse food crops.

**Plant-plastic-environment interactions:** Trophic transfer of microplastics up terrestrial food chains has been demonstrated, which has major implications on biomagnification and human exposure, ingestion, and health. Future research should elucidate the effect of microplastics on plant interactions investigating pathways of plastic ingestion by humans through food crops, elucidating differences between crop types and growing conditions.

## Research governance

We suggest that research in this emerging area would benefit from a scientific community initiative that sets standards for experimentation, similar to that for air pollution impacts and the critical loads concept (Bull, 1992). For plastics, we propose the code addresses four founding principles: (i) specify standardized mixtures (‘recipes’) of microplastic and nanoplastic types for experimentation, (ii) recommend the use of realistic concentrations to represent rural (0.1-0.5 mg/kg) and urban (900-5,000 mg/kg) environmental concentrations of plastics, (iii) encourage the development of dose-response experiments to establish critical thresholds, (iv) specify that long-term (whole growth cycle), rather than short-term experiments should have high priority. In this way, over a five-year period or so, significant advances in our understanding of plants and plastic pollution could be established.

A future that addresses these research and research governance gaps will help to quantify the impacts of plastic pollution in the terrestrial environment. Taken together, microplastics remain significantly under-studied forms of pollution in relation to plant performance, where increased knowledge is needed urgently to help mitigate their negative impacts, particularly for food crops consumed by humans.
