# Supplementary material for: Plants and microplastics: Growing impacts in the terrestrial environment
Source: Front Plant Sci. 2025 Sep 30;16:1666047. doi: 10.3389/fpls.2025.1666047 (PMC12518344; doi:10.3389/fpls.2025.1666047)
Supplement: Supplementary file 1 [file Table1.docx]

Supplementary Material

# Supplementary Figure

#
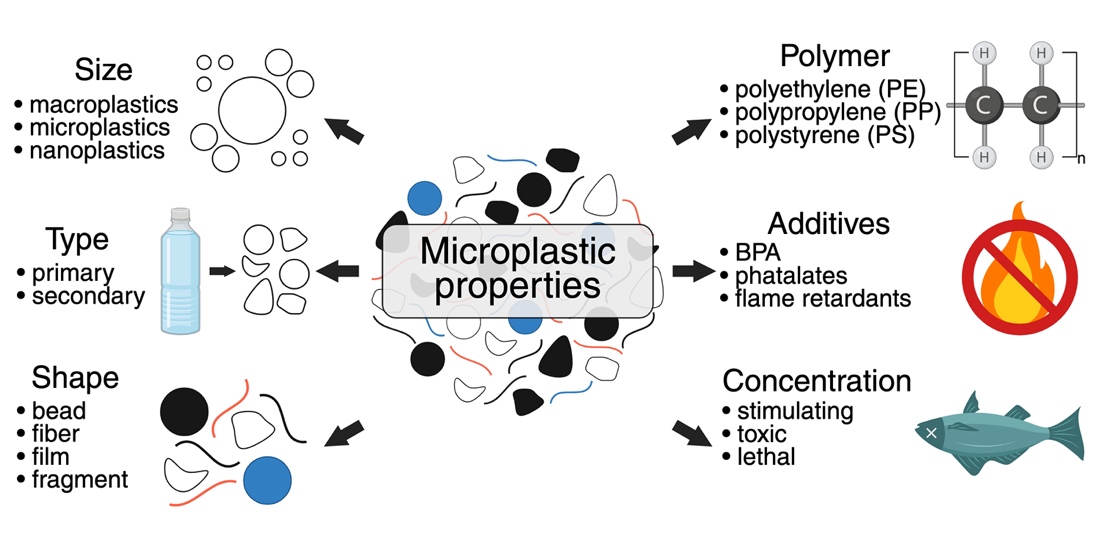


**Supplementary Figure 1:** **Diverse properties of microplastics that affect phytotoxicity.** Microplastics are diverse pollutants because they have different sizes, types, shapes, polymers, additives, and concentrations, which affect terrestrial plants. Abbreviations: bisphenol A (BPA). Created with BioRender.com.
